# NgLst8 Coactivates TOR Signaling to Activate Photosynthetic Growth in *Nannochloropsis gaditana*

**DOI:** 10.3390/microorganisms12122574

**Published:** 2024-12-13

**Authors:** Zhengying Zhang, Shu Yang, Yanyan Li, Dian Xie, Guobin Chen, Jiaxu Ren, Hongmei Zhu, Hantao Zhou

**Affiliations:** 1 State Key Laboratory of Marine Environmental Science, Xiamen University, Xiamen 361000, China; zhangzhengying@stu.xmu.edu.cn (Z.Z.); yangshummm@163.com (S.Y.); yanyanli2016@stu.xmu.edu.cn (Y.L.); xd1612819247@163.com (D.X.); cgb1251877642@126.com (G.C.); kivixxren@163.com (J.R.); 2 College of Ocean and Earth Sciences, Xiamen University, Xiamen 361000, China; flyzhu324@163.com; 3 State-Province Joint Engineering Laboratory of Marine Bioproducts and Technology, Xiamen University, Xiamen 361000, China

**Keywords:** *Lst8*, *Nannochloropsis gaditana*, TOR, photosynthesis, biomass

## Abstract

The target of rapamycin (TOR) serves as a central regulator of cell growth, coordinating anabolic and catabolic processes in response to nutrient availability, growth factors, and energy supply. Activation of TOR has been shown to promote photosynthesis, growth, and development in yeast, animals, and plants. In this study, the complete cDNA sequence of the *Lst8* gene was obtained from *Nannochloropsis gaditana*. The structure of *N. gaditana* LST8 comprises a typical WD40 repeat sequence, exhibiting high sequence similarity to several known LST8 proteins. By overexpressing the *Lst8* gene in *N. gaditana*, we constructed the NgLst8 transgenic algal strain and measured its photosynthetic activity and growth. We observed that an increase in LST8 abundance promotes the expression of TOR-related kinase, thereby enhancing photosynthetic growth. Transcriptome analysis further elucidated the response mechanism of elevated *Lst8* abundance in relation to photosynthesis. Our findings indicate that increased *Lst8* expression activates ABC transporter proteins and the MAPK signaling pathway, which regulate the transmembrane transport of sugars and other metabolites, integrate photosynthesis, sugar metabolism, and energy signaling, and modulate energy metabolism in algal cells through interactions with the TOR signaling pathway.

## 1. Introduction

The growth of organisms is influenced by a combination of environmental signals and is primarily constrained by nutrient availability. TOR is an evolutionarily conserved protein kinase and a key regulator of cell growth, sensing the nutrient status of the cell and transmitting signals to regulate cellular processes [1,2,3,4]. When essential nutrients are abundant, TOR signaling regulates cellular anabolism and catabolism to control cell growth processes [5,6,7,8]. In metazoans and fungi, TOR forms two distinct multiprotein complexes with different structures and functions, known as TOR complex 1 (TORC1) and TOR complex 2 (TORC2) [5,9]. These complexes were first identified in *Saccharomyces cerevisiae* and are highly conserved across eukaryotes [2,7,9,10,11].

The core components of TORC1 include TOR kinase, Raptor/KOG1, and LST8, while TORC2 is composed of TOR kinase, LST8, Sin1/AVO1, and Rictor/AVO3. In addition to TOR kinase, LST8 is a shared component in both TORC1 and TORC2, binding to the kinase domain of TOR to facilitate the catalytic activation of both complexes [12,13,14]. As a subunit of TOR, LST8 plays a critical role in vertebrate autophagy by promoting autophagosome formation and cellular degradation [15]. However, its functional role in invertebrates, particularly in immune regulation, remains largely unexplored. TORC1 in plants exhibits structural and functional conservation. In the model plant *Arabidopsis thaliana*, early studies showed that TOR is crucial for cell growth, and disruption of the TOR gene is lethal [16]. *A. thaliana* has two genes encoding LST8, but only one, LST8-1, is significantly expressed [1,17]. Although mutations in LST8-1 are not lethal, they result in stunted growth and delayed flowering [18]. A study further revealed that growth defects in *A*. *thaliana lst8-1* mutants can be rescued by mutations in the YAK1 kinase, suggesting that this kinase may play a crucial role in the plant TOR pathway by mediating stress signals [19].

Algae also possess TOR, and, similar to plants, the core proteins of TORC2, Rictor/AVO3 and Sin1/AVO1, are absent in algae [13,20,21]. In the model green alga *Chlamydomonas reinhardtii*, a large TORC1 complex has been identified, including homologs of TOR, Raptor, and LST8 [22,23,24]. Studies indicate that the LST8 protein in *C. reinhardtii* is functionally and structurally conserved [12,15,23]. Furthermore, *C. reinhardtii* LST8 has been found to bind to the kinase domain of TOR, and both proteins are part of a high-molecular-weight complex [4,25]. The effect of overexpressing TOR-related genes in algae, including red algae and green algae, on photosynthetic efficiency and the underlying mechanisms has been the subject of several studies [21,26,27]. Research has demonstrated that the TORC1 complex regulates metabolic and photosynthetic systems, contributing to algal adaptation to environmental stress [27]. Under high light or low CO_2_ conditions, algal LST8 regulates photoprotective mechanisms and CO_2_ fixation through the synthesis of photosynthetic pigments, the photosynthetic electron transport chain, and other related pathways [28]. This regulation enables the alga to better adapt to environmental changes.

*N. gaditana* is a marine unicellular alga with a long history of production and application. It has been widely used as an origin of algae for aquaculture feed and the production of eicosapentaenoic acid (EPA), which holds significant economic and industrial value. Additionally, its compact genome is amenable to genetic manipulation, facilitating metabolic engineering. The TOR pathway plays a crucial role in growth and lipid regulation, making it a key focus for research aimed at precisely controlling the growth and metabolism of *N. gaditana*. In this study, we obtained the full-length cDNA sequence of *Lst8* from *N. gaditana* and investigated its role in the photosynthetic growth of *N. gaditana* through overexpression. The structure of *N. gaditana* LST8 contains typical WD40 repeat sequences, which exhibit high similarity to several known LST8 proteins. The WD40 domain is one of the most abundant protein families in eukaryotes, often serving as a scaffold for assembling functional complexes through interactions with other macromolecules [29,30]. The WD40 repeat can form a stable β-propeller platform that facilitates the assembly of the mTOR complex or interactions with other proteins [23,31]. In this study, we aimed to elucidate the relationship between photosynthesis and the TOR signaling pathway in *N. gaditana*. By overexpressing the *Lst8* gene in *N. gaditana*, we measured the growth and photosynthetic activity of the overexpression strains and further explored the photosynthetic response mechanism through transcriptomic analysis of increased *Lst8* abundance in the TOR complex. Our results indicate that *Lst8* overexpression can activate ABC transporters and the MAPK signaling pathway, thereby regulating the transmembrane transport of metabolites such as carbohydrates. As shown in prior studies, the TOR pathway is tightly connected to nitrogen metabolism, as nitrogen availability influences the activation of transporters and downstream signaling pathways [32]. This suggests that regulation via ABC transporters not only mediates interactions between nitrogen metabolism and TOR signaling but also serves as a key link between photosynthesis and nutrient acquisition, thereby regulating broader metabolic processes, such as lipid biosynthesis. These findings underscore the complexity of the regulatory network governing algal growth and lipid production, where multiple pathways are interrelated and co-regulated, ultimately influencing metabolic efficiency under varying environmental conditions. Through these mechanisms, photosynthesis, nitrogen metabolism, and the TOR signaling pathway form a complex regulatory network that ensures algal cell proliferation and growth.

## 2. Materials and Methods

### 2.1. Culture Conditions and Microalgal Species

*N. gaditana* CCMP 526 was kindly provided by Danxiang Han (Institute of Hydrobiology, Chinese Academy of Sciences). The NgLst8 overexpression strain was generated through electroporation and exhibited resistance to zeocin at a screening concentration of 2 µg·mL^−1^.

*N. gaditana* was introduced into a marine water system (Fauna Marin, Holzger-lingen, Germany) enriched with F/2 media (G0154, Sigma-Aldrich, St. Louis, MO, USA). Each of the three replicates were cultured in 0.1 L medium, and the initial cell density was 1 × 10⁶ cells/mL. Cultures were maintained in an incubator at 1.5% CO_2_ concentration, 50 μmol m^−2^ s^−1^ illumination, 12 h light/dark cycle, and 22 °C temperature.

### 2.2. Phylogenetic Analysis and Multiple Sequence Alignment of the Lst8 Gene

Using *N. gaditana* as a query, we identified and screened the *Lst8* through BLAST (https://blast.ncbi.nlm.nih.gov/Blast.cgi; accessed on 9 November 2022) in eight eukaryotic species representing major taxa, including *A. thaliana*, *Dictyostelium discoideum*, *N. gaditana*, *Phaeodactylum tricornutum*, *Saccharomyces cerevisiae*, *C. reinhardtii*, *Thalassiosira pseudonana*, and *Cyanidioschyzon merolae*. Sequences were aligned and compared using MEGA5, followed by the construction of a phylogenetic tree. Transmembrane domains (TMDs) were predicted using InterPro (https://www.ebi.ac.uk/interpro/search/sequence/; accessed on 10 November 2022). We conducted a multiple sequence alignment analysis of representative species using EMBL-EBI (https://www.ebi.ac.uk/; accessed on 10 November 2022), visualized it with Jalview, and analyzed the results according to InterPro.

### 2.3. Vector Construction and Electrotransformation, DNA Extraction, and PCR Detection Transformation

The cloning of the *Lst8* gene (Naga_100004g35) from *N. gaditana* was based on the NCBI Sequence Library, using the conserved sequence of *Lst8* to perform a search in the EST Sequence Library. A conserved 1011 bp fragment was identified by sequence comparison. Primers specific to the *Lst8* gene were formulated from its sequence (see Table 1), yielding a full-length cDNA copy of this gene via PCR amplification. This DNA was subsequently employed in the construction of an overexpression vector. The pHSP plasmid contains the pHsp20 promoter and the zeocin resistance gene [33]. The amplified fragments were ligated using Gibson assembly, transformed into *Escherichia coli*, and screened on LB+Amp plates. The recombinant plasmid was extracted and named pHSP-Lst8-TUB-Zeocin.

To generate transgenic strains overexpressing *Lst8*, electroporation was set at 2200 V, 50 μF, and 600 Ω [33,34]. Following electroporation, the algal cells were moved to 15 mL of marine water and cultured under low-light conditions at 22 °C for 48 h. After centrifugation at 4000 rpm for 15 min, cells were spread on plates with 2 μg/mL zeocin and incubated at 22 °C in 50 μmol m^−2^ s^−1^ light.

Samples underwent total DNA extraction via an efficient plant DNA extraction kit (GeneBetter^®^, Beijing, China). Primers (listed in Table 1) validated the amplifications of *Lst8* and resistant zeocin fragments within the transgenically modified *N. gaditana* genome. The PCR protocol involved two initial steps: 30 s at 94 °C, then 35 cycles each, comprising 10 s at 98 °C, 30 s at 55°C, ending with 1 min at 72 °C. The amplified products were visualized electrophoretically on 1% agarose gel.

### 2.4. Identification of Microalgal Transformants at the Molecular Level

Microalgal transformants were collected by filtering through a 0.22 μm PCM (Millipore, Isopore^TM^, Billerica, MA, USA). The samples were then transferred to 2 mL grinding tubes with 2 mL of PBS buffer and centrifuged at 6000 rpm for 10 min at 4 °C. After the samples were frozen in liquid nitrogen, the algal cells and prechilled beads were placed in the grinder and ground at 60 Hz for 30 s. Following grinding, the RNA was extracted using an RNA extraction kit (Genebetter, Beijing, China).

The expression levels of the target genes were confirmed using RT-PCR with specific primers, and gene expression was evaluated in detail (Supplementary Table S3). The following program was used: 95 °C for 30 s, followed by 40 cycles of 95 °C for 5 s and 60 °C for 30 s. The 2^−ΔΔCT^ method was applied, with the universally conserved *actin* gene serving as the reference for normalization [35].

### 2.5. Western Blotting

During exponential growth, a mix of overexpressed and wild-type algae is lysed via the addition of 200 μL cell lysis buffer (87787, Thermo, Waltham, MA, USA) and 1 mM protease inhibitor. Total protein is isolated via a 20 min centrifugation at 4 °C, 16,000 rpm. This is quantified using the BCA method (P0010SN, Beyotime, Shanghai, China) and then subjected to SDS-PAGE. Then, proteins are moved onto a PVDF membrane for 1 h at 100 V. The membrane is washed thrice with 1× TBST before incubation in a 5% skim milk/1× TBST solution at 37 °C for 1 h. A rabbit anti-FLAG tag antibody (LABLEAD, Beijing, China), diluted to 1:1000, confirms FLAG-tagged NgLst8 expression, followed by a secondary rabbit anti-antibody detection step. The Chemi Doc system (Bio-Rad, Hercules, CA, USA) and Chemiluminescence (ECL) are employed for signal visualization.

### 2.6. Cell Growth

The growth assessment involved cellular density (cells/mL) and dry cell weight (DCW) measurement. An initial amount of 10^6^ *N. gaditana* cells/mL were cultured using a flow cytometer (Beckman, CytoFlex S, Brea, CA, USA). The cell count was obtained from their auto-fluorescent properties at 488 nm illumination with 685 nm emitted photons on a fluorospectrometer (CytoFlex). Particle sizes and chlorophyll fluorescence exams were achieved on a similar device armed with CytExpert 2.5 software. The 488 nm laser enabled FSC-A channel particle sizing, while the 690 nm/50 BP channel measured chlorophyll fluorescence for single-cell chlorophyll content determination.

### 2.7. Biomass Measurements and Lipid Content

At the end of the light cycle on the 12th day of cultivation, an equal volume of the transformed algal strain was collected and centrifuged at 5000× *g* for 10 min compared to the control *N. gaditana* culture. The algal cells were washed three times with deionized water to remove salts and then collected in pre-weighed, clean, dry centrifuge tubes. Following drying, the weight was measured on an electronic balance (Sartorius, Quintix^®^, Göttingen, Germany) with an accuracy of 0.1 mg.

The extraction of total lipids began with freeze-dried samples, which were chopped as finely as necessary to form a powder. After grinding, 10 mL of a methanol, chloroform, and formic acid mix (20:10:1 *v*/*v*) was added to the extract and stirred vigorously for 10 min in a vortex mixer. Then, 3 mL of a phosphoric acid/potassium chloride blend (0.2 M H_3_PO_4_, 1 M KCl) was incorporated and agitated for 2 min prior to spinning for 15 min at 5000 rpm. The chloroform layer was blown dry with nitrogen after transfer to a 15 mL test tube. Lipid quantification utilized the following equation: Lipid content (% DCW) = B/DCW (B representing the total lipid, DCW denoting the dry cell weight of the sample).

### 2.8. Measurement of Chlorophyll Fluorescence

The chlorophyll fluorescence parameters of *N. gaditana* were measured using a MULTI-COLOR-PAM (MULTI-COLOR-PAM, Walz, Effeltrich, Germany). We mainly evaluated the following PSII chlorophyll fluorescence parameters: Fv/Fm, Y(II), and rETR. Algal samples selected from batch cultures were diluted to approximately 300 μg/L chlorophyll concentration and exposed to a 20 min light treatment. The instrument underwent calibration in advance of the experimental measurements. In preparation for the experimental measurements, samples were positioned within cuvettes, with the Ft value set to 1.5. The measuring light (ML) was adjusted to a wavelength of 440 nm, while the actinic light (AL) equated to the white light intensity utilized for the cultivation phase. Specific steps included placing 2.0 mL of the sample into a 10 mm diameter cuvette to measure the chlorophyll fluorescence of the algae. First, the minimum fluorescence (Fo) was recorded in low-light environments. Subsequently, the maximum fluorescence (Fm) was ascertained via saturation pulses. During the light adaptation process, the actinic light was turned on, allowing for a 20 s interval between saturating pulses. Maximum light-adapted fluorescence (Fm′) and steady-state fluorescence (F) were documented during this process. The Fv/Fm and actual Y(II) were calculated using the following formulae: Fv/Fm = (Fm − Fo)/Fm and Y(II) = (Fm′ − F)/Fm′.

The wavelength of the measurement light was set at 440 nm, and 20 levels of light intensity were established to determine the rETR and the rapid light response curves of PSII. White light was used as the actinic light within the wavelength range of 420–640 nm, with each light intensity level maintained for 20 s, followed by a saturating pulse lasting 800 ms upon completion of each light intensity phase. The formula for calculating the relative rETR at each light intensity involves rETR (II) = Y(II) × PAR × 0.5, where PAR denotes the actinic light intensity, and the coefficient 0.5 assumes that the absorbed photons are evenly distributed between photosystems I and II. The rETR values can be modeled according to the following formula: rETR = PAR/(a × PAR2 + b × PAR + c), where PAR is the specific light intensity, and a, b, and c are the fitted parameters. Based on the light response curve, the maximum relative values of rETRmax and Ik were derived by using the equations proposed by Eilers and Peeters (1998): rETRmax = 1/(b + 2 × (a × c)1/2) and Ik = c/(b + 2 × (a × c)1/2) [36].

### 2.9. Transmission Electron Microscopy Analysis

On the third day of batch culture, samples were collected at the end of the light cycle. *N. gaditana* cells were centrifuged at 3000× *g* for 5 min, then resuspended in 0.1 M phosphate buffer (pH 7.4) and fixed with 4% glutaraldehyde. The samples were kept in the dark at 4 °C overnight for fixation. Sample preparation and sectioning were performed by the Shared Platform for Biomedical Instruments at Xiamen University. High-resolution transmission electron microscopy (TEM) images were obtained using a Hitachi HT-7800 (Hitachi, Tokyo, Japan) at 100 kV. Based on the TEM images, 15 intact cells from each sample were selected for size analysis using ImageJ software (ImageJ 1.53t, 2023, Bethesda, MD, USA).

### 2.10. RNA-Seq Library Preparation for Transcriptome Sequencing

Samples utilized 1 μg RNA for RNA preparation. Using the Yeasen Biotech Co., Ltd., Shanghai, China’s Hieff NGS Ultima dual-mode mRNA library prep kit, sequencing libraries were produced per manufacturer guidelines. mRNA purification from total RNA utilizing poly (T)-attached magnetic beads proceeded with subsequent syntheses of both first- and second-strand cDNA. Exonuclease/polymerase treatment converted overhangs to blunt ends prior to adenylating the DNA fragments’ 3′ ends. Hair-pin-structured NEBNext adapters were ligated after purification via the AMPure XP system. USER enzyme (NEB, Ipswich, MA, USA), 3 μL in volume, was then added to the ligated cDNA of specific size, incubated for 15 min at 37 °C, 95 °C for 5 min, and subjected to PCR. PCR amplification used Phusion high-fidelity DNA polymerase, universal PCR primers, and index (X) primers. The final purification of PCR products (AMPure XP system) and the assessment of library quality on the Agilent Bioanalyzer 2100 system concluded this process.

### 2.11. Transcriptome Assembly, Annotation, and Differential Expression Analysis

The raw reads were processed using the BMKCloud bioinformatics pipeline tool on the online platform (www.biocloud.net). Initially, the raw data in FASTQ format were processed using custom Perl scripts to eliminate adapter sequences, poly-N sequences, and low-quality reads, ultimately generating clean data. Subsequent analyses relied exclusively on these high-quality clean data. To further refine the dataset, adapter sequences and low-quality reads were removed. The reference genome set was derived from the annotated *N. gaditana* B-31 genome (http://www.nannochloropsis.org/page/ftp/; accessed on 9 February 2024) and served as the basis for subsequent read analysis and annotation, allowing only perfectly matched or single-mismatched reads. The reference genome was aligned using HISAT2 software (HISAT2 2.2.1, 2023, Baltimore, MD, USA). Gene functions were annotated through multiple databases, including Nr (NCBI non-redundant protein sequences), Pfam (protein families), KOG/COG (clusters of orthologous groups), Swiss-Prot (manually annotated and reviewed protein sequence database), KO (KEGG ortholog database), and GO (gene ontology). Differential expression analysis between the two conditions/groups was conducted using DESeq2, which employs statistical methods based on a negative binomial distribution to identify differentially expressed genes in the count data. For KEGG pathway enrichment, the KOBAS database (KOBAS 3.0, 2015, Beijing, BJ, China) and clusterProfiler software (clusterProfiler 4.6.2, 2023, Shanghai, SH, China) were used to statistically evaluate the enrichment of differentially expressed genes (DEGs). Additionally, GO enrichment analysis of DEGs was performed using the clusterProfiler package (clusterProfiler 4.6.2, 2023, Shanghai, SH, China), utilizing the Wallenius non-central hypergeometric distribution to correct for gene length biases among the DEGs.

### 2.12. Statistical Analysis Methodology

In parallel with statistical analysis of the data generated in this study, biological and technical replication analyses were performed, and in order to verify the reproducibility of the observed tendencies, 3 bioreplicates were analyzed for each growth condition, and 3 techniques were repeated for each bioreplicate. The mean value for each data point was calculated from these technical replicates. All statistical analyses were performed using GraphPad Prism software (GraphPad Prism 8.4, 2020, Boston, MA, USA) with two-way ANOVA. Technical data from all individual bioreplicates were incorporated for the analysis of variance (ANOVA). The error bar represents the standard deviation of the three sample means. The treatment effect was evaluated using the Tukey test (*p* < 0.05). 

## 3. Results

### 3.1. Transformation Vector Construction of NgLst8

To investigate the *Lst8* gene across different species, we used the *Lst8* gene sequence of *N. gaditana* and searched for homologs in various eukaryotes using BLAST (https://blast.ncbi.nlm.nih.gov/Blast.cgi; accessed on 9 November 2022). We then constructed a phylogenetic tree of *Lst8* from the sequences obtained. For comparison of LST8, we specifically selected species that represented important evolutionary milestones or had unique physiological characteristics. This approach aimed to identify specific amino acid structures associated with changes in enzyme activity and regulation across these species while also obtaining comprehensive and high-quality LST8 sequences from public databases. The results indicated that the *Lst8* gene in *N. gaditana* shares a high degree of homology with those in other species, and it is found not only in terrestrial plants but also in a wide range of micro-organisms. We conducted a multiple sequence alignment analysis of representative species (Supplementary Figure S1). The *Lst8* gene of *N. gaditana*, which consists of seven WD40 motifs, shares high similarity with the *Lst8* sequences of *P. tricornutum* and *T. pseudonana*.

**Figure 1:**
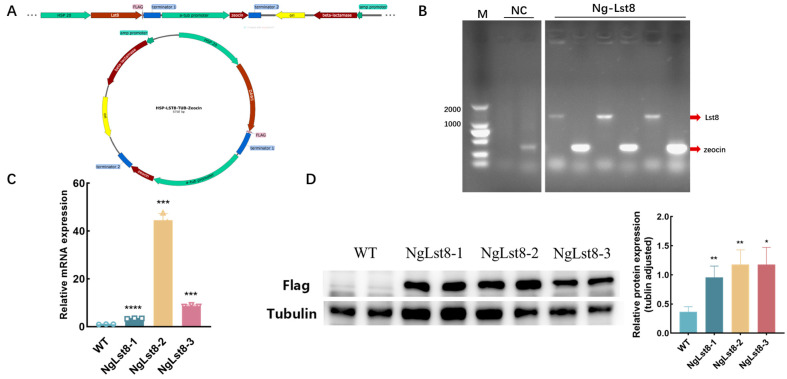
Construction and validation of the NgLst8 transformants. (**A**) Modeling of the NgLst8 transformants. (**B**) Electrophoretic verification results of the *Lst8* and *zeocin* fragments. NC: negative control (A vector lacking the target gene LST8, containing only the zeocin resistance gene, to serve as a negative control). (**C**) The RT-qPCR of NgLst8 and WT strains. (**D**) The Western Blotting of NgLst8 and WT strains. **** *p* < 0.0001, *** *p* < 0.001, ** *p* < 0.01, * *p* < 0.05. The error bars represent the standard deviation calculated from three replicate measurements.

The recombinant plasmid constructed using the *Lst8* gene was named pHSP-Lst8-TUB-Zeocin (Figure 1A). Transgenic algal strains overexpressing *Lst8* were subsequently constructed via electrotransformation. Gene-specific primers (Table 1) were used to amplify the *Lst8* and *zeocin* fragments with homologous arms from the plasmid, using the transformed genome as a template. Electrophoretic validation showed that the relative molecular weights of the bands were 1056 bp and 375 bp, respectively, which matched the expected sizes, indicating successful integration of the gene sequences into *N. gaditana* (Figure 1B). Transcript levels of the overexpressed *Lst8* gene in the transgenic algal strains were further assessed by qPCR. Three overexpression strains, named NgLst8-1, NgLst8-2, and NgLst8-3, showed significantly elevated *Lst8* expression levels, which were 3.42-fold, 44.5-fold, and 8.7-fold greater than the wild type (WT) (Figure 1C). Protein was extracted from the algal strains and diluted to the same concentration for Western Blot analysis. The results (Figure 1D) indicated that the tubulin antibody served as the internal reference, and the overexpressed protein band was approximately 37 kDa, while no corresponding band was observed in the WT. These results confirm that the NgLst8 overexpression plasmid was successfully integrated into the genome of *N. gaditana*, leading to normal transcription and translation, and that the recombinant protein was successfully expressed in *N. gaditana*.

### 3.2. Effect of Overexpression of the Lst8 Related to Growth and Photosynthetic Rate

*N. gaditana* primarily grows through photosynthesis, and its final biomass accumulation directly reflects photosynthetic efficiency. Therefore, growth and biomass production traits were investigated. To evaluate the growth performance of the overexpressed strains, three transgenic algal strains (NgLst8-1, NgLst8-2, and NgLst8-3) were cultured in seawater medium to determine their growth phenotypes.

**Figure 2:**
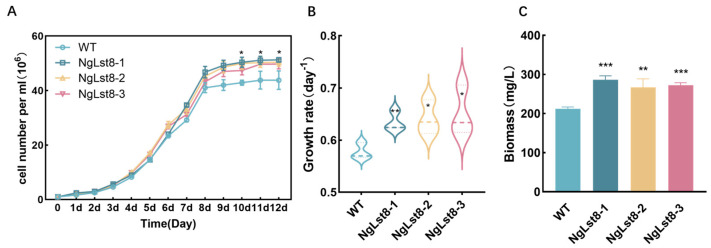
Growth characteristics of NgLst8 strains. (**A**) Growth profile of the WT and the NgLst8 strains. (**B**) Growth ratios from day 3 to day 4. (**C**) Biomass on day 12 for NgLst8 and WT. The error bars represent the standard deviations derived from three biological replicates. * *p* < 0.05, ** *p* < 0.01, *** *p* < 0.001.

First, the difference in cell growth between the NgLst8 and the WT strains was assessed using flow cytometry over a 12-day culture period. In contrast, the overexpressed algal strain NgLst8 grew with higher cell numbers than the WT in all cases (Figure 2A). The cell numbers of the overexpressed algal strain population were significantly higher than those of the WT (43.81 × 10^6^ cells /mL (*p* < 0.01)) when the cell numbers of NgLst8 were 51.27 × 10^6^ cells /mL, 50.41 × 10^6^ cells /mL, and 49.73 × 10^6^ cells/mL, respectively, from the culture to 12 days. Based on our preliminary findings, we measured growth rates from day 3 to day 4 to observe early trends and identify initial changes before larger differences became apparent at later time points. The growth rates from culture to the logarithmic phase were measured, and the average daily growth rates of overexpressed algal strain NgLst8 were 0.635 day^−1^, 0.642 day^−1^, and 0.651 day^−1^ from day 3 to day 4, respectively, compared with 0.577 day^−1^ (*p* < 0.05) for the WT (Figure 2B), with an average (*p* < 0.01) increase of 11.4%. Meanwhile, the dry weight of biomass of NgLst8 on day 12 of batch culture was determined in this experiment (Figure 2C). The results showed that the cell dry weights of NgLst8 were 286.56 mg/L, 270 mg/L, and 272.89 mg/L on day 12 of batch culture, which was significantly higher than that of the WT at 212.2 mg/L (*p* < 0.01). These results suggest that *Lst8* overexpression in *N. gaditana* promotes cell growth and biomass accumulation.

Oxygen-producing photosynthetic organisms absorb and transmit light energy through chlorophyll attached to the chloroplast-like vesicle membrane. Therefore, the chlorophyll content in algal cells reflects their ability to absorb and capture light. In this study, the differences in cellular chlorophyll content were assessed via flow cytometric determination of the chlorophyll fluorescence values of NgLst8 and WT strains. Chlorophyll fluorescence values were measured from single cells on the third day of the logarithmic growth phase of batch culture. The results indicated that the single-cell chlorophyll fluorescence value of NgLst8 increased by an average of 15.81% (*p* < 0.01) compared to WT (Supplementary Figure S2A).

Light energy utilization is an index used to characterize the efficiency of photosynthetic organisms in converting atmospheric CO_2_ into biomass through photosynthesis and serves as an important parameter for evaluating photosynthetic efficiency. To precisely compare the light energy utilization rate of *N. gaditana* under different culture conditions, light energy utilization was obtained as PCE = DW × Q / (PAR × T × S × E) (Supplementary Figure S2B) [37]. The biomass content of *N. gaditana* was determined under 50 μmol photons m^−2^ s^−1^, and the light energy utilization of the overexpressed strain NgLst8 was significantly higher than that of the WT, with an average increase of 30.3%. These findings indicate that NgLst8 can efficiently utilize light energy under medium-light conditions.

### 3.3. Effect of NgLst8 Overexpression on Photosynthetic Characteristics During Photoautotrophic Growth

**Figure 3:**
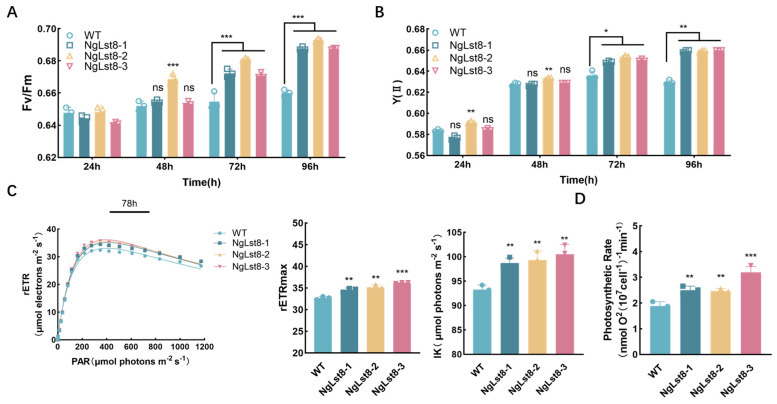
Chlorophyll fluorescence parameters of PSII of NgLst8 and WT strains. (**A**) Fv/Fm of NgLst8 and WT strains. (**B**) Y(II) of NgLst8 and WT strains. (**C**) Relative photosynthetic electron transfer efficiency (rETR), rETRmax, and IK of WT and NgLst8 strains. (**D**) Photosynthetic oxygen release rate of WT and NgLst8 strains. *** *p* < 0.001, ** *p* < 0.01, * *p* < 0.05; ns indicates *p* > 0.05. The error bars indicate the standard error of three biological replicates.

As a photosynthetic micro-organism, photosynthesis directly impacts the biomass and organic matter synthesis of *N. gaditana*. To determine the effect of *Lst8* overexpression on the photosynthetic activity of *N. gaditana*, chlorophyll fluorescence parameters, including the maximum photochemical efficiency (Fv/Fm), actual photochemical efficiency (Y(II)), and the relative electron transport rate (rETR) in photosystem II (PSII) during the mid-photocycle from day 1 to day 4, were measured in this study. The results indicated that the Fv/Fm values of the overexpressing algal strains NgLst8-1, NgLst8-2, and NgLst8-3 were all increased compared to the WT. At the midpoint of the photoperiod on day 3 (72 h) (Figure 3A), the Fv/Fm values of NgLst8-1, NgLst8-2, and NgLst8-3 were 0.672, 0.681, and 0.671, respectively, whereas the Fv/Fm value of the WT was 0.654. These values were significantly higher (*p* < 0.05) than those for the WT. The actual photochemical efficiencies Y(II) of NgLst8-1, NgLst8-2, and NgLst8-3 were 0.649, 0.654, and 0.651, respectively, compared to 0.636 for the WT (Figure 3B). This indicates that the Fv/Fm and Y(II) values of the overexpressing NgLst8 strains during the logarithmic growth phase were significantly higher (*p* < 0.05) than those of the WT, enhancing the efficiency of the photosynthetic apparatus for light energy conversion.

Light response curves are commonly used to assess the adaptation of microalgae to different light intensity conditions. The rETR of PSII at the mid-photoperiod on day 3 (78 h) of incubation was measured and fitted to obtain a curve (Figure 3C). At light intensities below 162 μmol photons m^−2^ s^−1^, the curves of the NgLst8 and WT nearly overlapped. However, when the light intensity exceeded 162 μmol photons m^−2^ s^−1^, the rETR values of the three strains of NgLst8 were higher than those of the WT (*p* < 0.05). The maximum rETR (rETR max) values for NgLst8-1, NgLst8-2, and NgLst8-3 were 34.5, 35.2, and 36.1 μmol electrons m^−2^ s^−1^, respectively, compared to 32.6 μmol electrons m^−2^ s^−1^ for the WT. The half-saturation light intensity (Ik) of NgLst8-1, NgLst8-2, and NgLst8-3 was 98.73, 99.33, and 100.5 μmol photons m^−2^ s^−1^, respectively, compared to 93.26 μmol photons m^−2^ s^−1^ for the WT (Figure 3C). These findings indicate that the overexpressing strains of NgLst8 exhibited higher rETRmax and Ik values. The photosynthetic oxygen release rate is a key indicator of the photosynthetic carbon sequestration capacity of *N. gaditana* under optimal light conditions. Overexpression of the *Lst8* gene increased the photosynthetic oxygen release rate of *N. gaditana* by an average of 45% compared to the WT (Figure 3D).

These results suggest that *Lst8* overexpression enhances the photosynthetic rate of PSII, resulting in higher light energy conversion efficiency and photosynthetic capacity in the transgenic algal strains. Additionally, these strains demonstrated a heightened response to changes in light intensity and exhibited greater photosynthetic potential.

### 3.4. The Chloroplast Morphology of NgLst8

**Figure 4:**
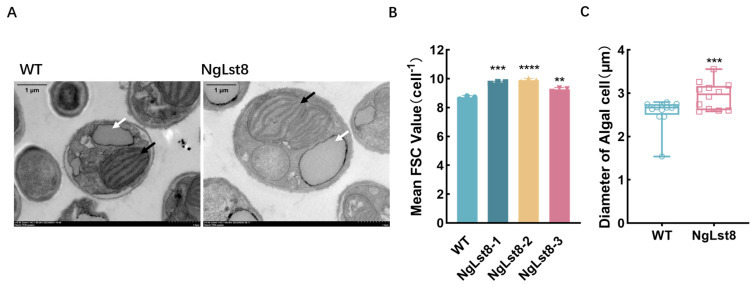
Chloroplast morphology and cell grain size of NgLst8. (**A**) Transmission electron microscopy of WT and NgLst8 strains on day 3. White arrows point to lipid droplets and black arrows to chloroplast. (**B**) FSC of WT and NgLst8 strains on day 3. (**C**) Diameter of algal cells after three days of WT and NgLst8 strains. **** *p* < 0.0001, *** *p* < 0.001, ** *p* < 0.01. The error bars indicate the standard error of three biological replicates.

Chloroplast formation and development are crucial molecular processes for photosynthetic organisms, such as algae and plants, to carry out photosynthesis, serving as the foundation of algal photosynthesis. To further investigate whether the enhanced in photosynthetic autotrophy due to TOR activation is related to chloroplast development, we observed and analyzed chloroplast formation using transmission electron microscopy (TEM) with WT and overexpressing algal strain NgLst8. Previous experiments revealed that the transformed strain exhibited a stable physiological phenotype, particularly in growth and photosynthesis-related parameters. Based on this stability, we randomly selected one algal strain for further evaluation of its cellular properties. The results showed that after 3 days of growth in normal culture (ASW + F/2), both WT and NgLst8 strains exhibited intact chloroplast structures (Figure 4A). However, chloroplast division was accelerated in the NgLst8 strain (black arrows). The cellular lipid droplets were not affected by the accelerated chloroplast division (white arrows). This suggests that overexpression of the *Lst8* gene had dual effects: it promoted chloroplast division, leading to an increase in the final biomass, while not significantly altering the lipid content or reducing the size of lipid droplets. This indicates that overexpression did not inhibit lipid synthesis despite enhanced chloroplast development. However, the increase in biomass due to chloroplast expansion ultimately resulted in a higher lipid yield (Supplementary Figure S3).

Biomass is determined by the product of individual mass and population size, making the average single-cell mass an important factor in the overall biomass of *N. gaditana*. At a constant density of cellular contents, cell volume directly influences single-cell mass, resulting in a positive correlation between cell volume and biomass. The forward scatter (FSC) values of the overexpressing strain and the WT were measured using flow cytometry, and cell size was also assessed using TEM. The results showed that on day 3 of culture, the average single-cell size of NgLst8 was 2.94 μm, whereas that of the WT was 2.56 μm (Figure 4C). The average single-cell size of the overexpressing strain NgLst8 was significantly larger (*p* < 0.05) than that of the WT, consistent with the FSC values obtained from flow cytometry (Figure 4B).

These results indicate that overexpression of the *Lst8* gene contributes to chloroplast formation and division in *N. gaditana*, affecting chloroplast proliferation, enlarging cell size, and accelerating photosynthetic autotrophic growth.

### 3.5. Enhanced TOR Signaling and Photosynthetic Signaling in NgLst8

**Figure 5:**
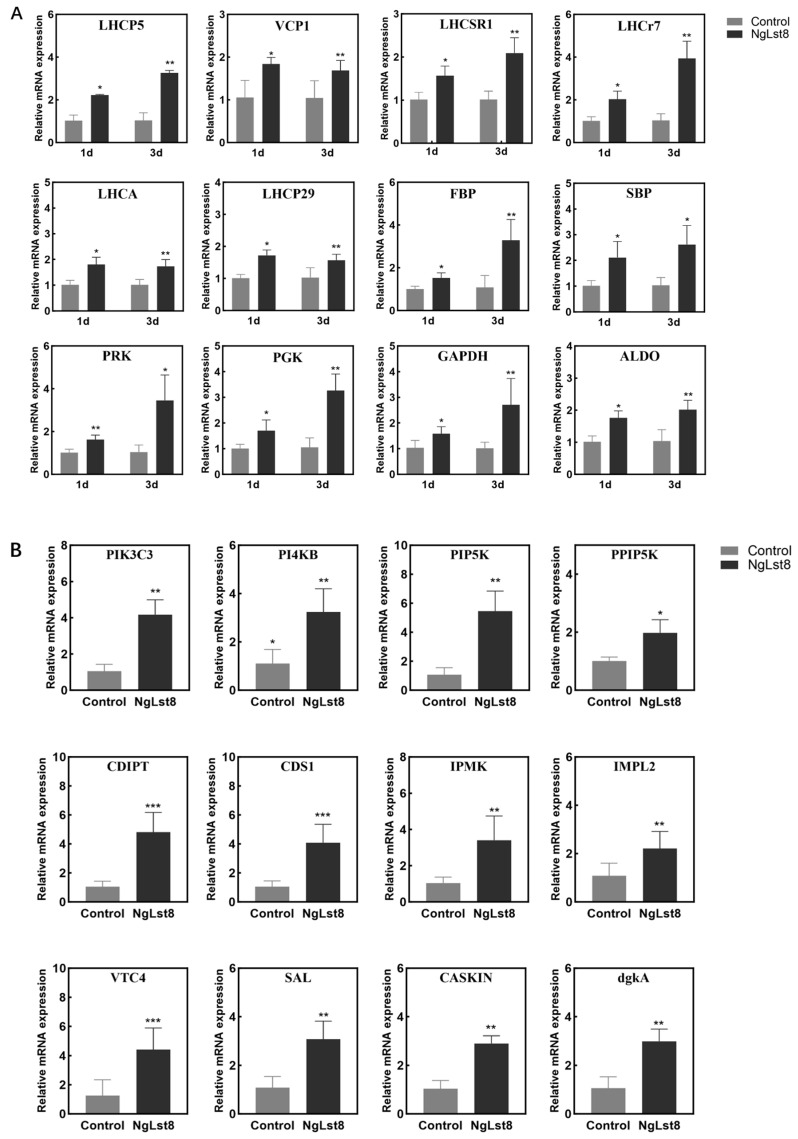
Transcription of WT and NgLst8 strains’ related genes. (**A**) Transcription of WT and NgLst8 strains’ photosynthesis-related genes. (**B**) Transcription of WT and NgLst8 strains’ phosphatidylinositol pathways. *** *p* < 0.001, ** *p* < 0.01, * *p* < 0.05. The error bars indicate the standard error of three biological replicates.

Photosynthesis is a critical physiological process in photosynthetic organisms such as microalgae, providing the energy and sugars essential for autotrophic growth. Transcriptome data analysis in previous studies has revealed a strong link between the TOR signaling pathway and photosynthetic signaling [38,39]. To further investigate the molecular mechanism between *Lst8* overexpression, TOR signaling, and photosynthetic autotrophic growth, we analyzed the expression of photosynthesis-related marker genes using qRT-PCR after 1 and 3 days of normal incubation with NgLst8 (Figure 5A). These genes play key roles in photosynthetic light reactions, carbon fixation, and chlorophyll synthesis and degradation in *N. gaditana*. Specifically, *LHCr7* (Naga_100641g3), *LHCP5* (Naga_100027g19), and *VCP1* (Naga_100012g50) are crucial for light reactions, while *LHCSR1* (Naga_100173g12), *LHCA* (Naga_100056g42), and *LHCP29* (Naga_100168g14) are involved in photosynthetic processes. The key rate-limiting enzyme genes involved in carbon fixation include *FBP* (Naga_101891g1), *SBP* (Naga_100049g1), *PRK* (Naga_100157g10), *PGK* (Naga_100410g3), *GAPDH* (Naga_100081g16), and *ALDO* (Naga_100154g7).

The phosphatidylinositol (PI) signaling pathway regulates cell membrane dynamics and vesicular transport while also participating in cell growth and metabolism through interactions with other key signaling pathways [40]. In particular, PI plays a crucial regulatory role in the TOR signaling pathway [2]. PI signaling ensures efficient cellular utilization of photosynthetic products by regulating membrane transport and energy metabolism [41,42]. The sugars produced by photosynthesis serve as energy signals that activate the PI3K-TOR pathway, promoting protein synthesis, cell division, and growth. Additionally, PI supports photosynthetic metabolism by regulating vesicular transport, ensuring material exchange and energy flow between organelles [39,43,44]. We analyzed the expression of marker genes in the PI signaling pathway using qRT-PCR, and the results showed that almost all PI pathway genes were upregulated in NgLst8 (Figure 5B).

The quantitative analysis indicated that nearly all genes involved in the photosynthetic response and chlorophyll synthesis were upregulated in NgLst8. These results suggest that overexpression of the *Lst8* gene enhances phosphatidylinositol signaling, mediates TOR signaling, and regulates the expression of photosynthesis-related genes, thereby contributing to the photosynthetic growth of *N. gaditana*.

### 3.6. Transcriptome Reveals Potential Response Mechanism of TOR Signaling Activation via Overexpression of the Lst8

The *Lst8* gene is a key component of the TOR complex, maintaining the integrity and functionality of the TORC1 complex through direct binding to TOR kinases. Overexpression of *Lst8* enhances the stability and activity of the TOR complex, thereby activating downstream signaling processes. To further investigate the molecular mechanism by which NgLst8 mediates the TOR complex and promotes photosynthetic growth in *N. gaditana*, RNA-seq analysis was conducted to examine gene expression differences between WT and NgLst8 strains, aiming to elucidate potential response mechanisms.

Since the qRT-PCR results at the onset of the photoperiod indicated that the expression of the NgLst8 photosynthetic gene was activated, we aimed to explore the pathway response after photosynthesis activation. Samples of NgLst8 were collected at the end of the photoperiod on the third day of incubation and compared to the control. The reference genome used for transcriptome annotation in this study was *N. gaditana* (NCBI species classification number: 72520). A total of 39.54 Gb of clean data was obtained from the samples, and the percentage of Q30 bases exceeded 92.71%, with each sample reaching 6.44 Gb of clean data, indicating sufficient sequencing depth and good quality (Supplementary Table S1). Principal component analysis (PCA) of transcriptome samples showed good clustering within replicates, with distinct separation between groups, demonstrating high repeatability and clear differences between conditions (Supplementary Figure S4). The transcriptome analysis focused on differentially expressed genes (DEGs), revealing 519 DEGs, of which 488 were significantly upregulated, accounting for over 90% of the total (Supplementary Figure S4). Ten DEGs were randomly selected for qRT-PCR analysis to validate the accuracy of the transcriptome data. The qRT-PCR results were consistent with the transcriptome trends, confirming the reliability of the data (Supplementary Figure S5).

**Figure 6:**
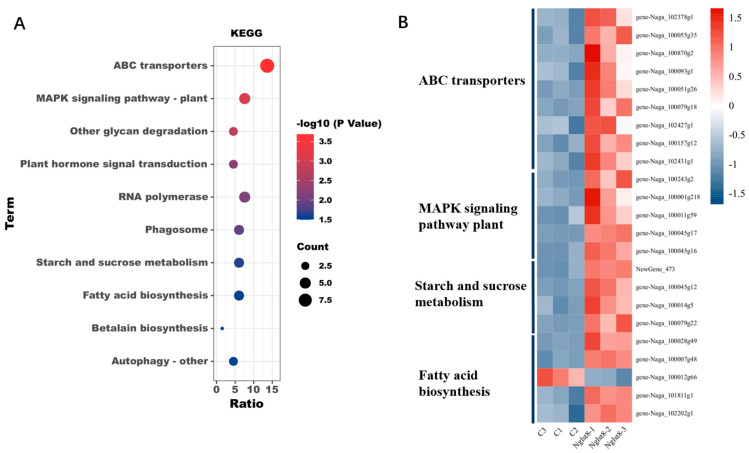
The transcriptomic analysis of WT and NgLst8 strains. (**A**) KEGG enrichment analysis of WT and NgLst8 strains. (**B**) Heatmap of differential gene enrichment pathways.

At the end of the photoperiod, the sequencing results revealed 519 differentially expressed genes (DEGs), of which 488 were upregulated and 31 were downregulated. KEGG pathway analysis showed significant enrichment in pathways such as “ABC transporters”, “MAPK signaling pathway—plant”, “Plant hormone signal transduction”, “Starch and sucrose metabolism”, “Fatty acid biosynthesis”, and “Betalain biosynthesis”, among others, which were all significantly upregulated (Figure 6A). Further analysis of the DEGs revealed significant upregulation of genes related to starch and sucrose metabolism, mainly involved in the degradation of cellulose into polysaccharides (Figure 6B; details in Supplementary Table S2). In particular, the upregulation of fatty acid synthase (FASN) in fatty acid biosynthesis may promote the synthesis of long-chain fatty acids, which serve as a major source of cellular energy and produce adenosine triphosphate (ATP) to provide energy for cellular functions. Additionally, FASN plays a regulatory role in signaling molecules, receptor proteins, and ion channels, thereby affecting cell signaling (Figure 6B). Significant upregulation was also observed in genes encoding ABC transporter proteins ABCA3, ABCB1, and ABCG2 (Figure 6B). The ATP-binding cassette (ABC) transporter family represents ATP-hydrolysis-dependent transporters that play a crucial role in regulating metabolite transport across membranes and nutrient redistribution. During photosynthesis, the sugars produced act as primary activators of the TOR pathway, and activation of TOR signaling further regulates cell growth, metabolism, and protein synthesis, forming a link between photosynthesis and cell growth. Starch and sucrose metabolism can synergize with TOR signaling by providing sugars, which, on the one hand, sense light and nutrient signals via the MAPK signaling pathway and, on the other hand, participate in TOR activation through ABC transporter proteins, thus regulating the transmembrane transport of metabolites such as sugars. Furthermore, MAPK6 expression was significantly upregulated in the “MAPK signaling pathway—plant” and “Plant hormone signal transduction” pathways. In *C. reinhardtii*, the MAPK signaling pathway regulates nitrogen assimilation and, through interactions with other signaling networks, helps the alga adjust its cell growth and metabolic state in response to environmental stresses. Thus, we hypothesize that MAPK6 may play a broad role in regulating additional metabolic processes in microalgae, including those involved in photosynthesis and overall cell growth [45,46]. The light and sugar signals produced during photosynthesis, similar to nitrogen signals, can regulate TOR signaling through the MAPK pathway, thereby influencing plant metabolism and growth. Therefore, it is hypothesized that upregulation of the MAPK6 gene triggers a signaling cascade to activate downstream pathways, promoting cell proliferation, differentiation, and growth. Together, photosynthesis and TOR signaling constitute a complex regulatory network that ensures plant growth and metabolic regulation in variable environments (Figure 6B).

**Figure 7:**
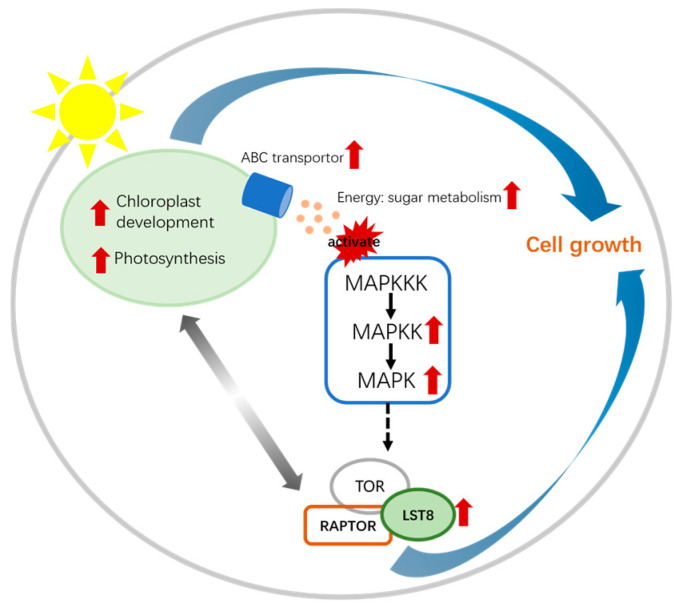
The transcriptome reveals a model in which overexpression of the *Lst8* gene regulates photosynthetic growth in *N. gaditana.* The red arrows indicate upregulation of expression. Black arrows represent direct effects, gray arrows indicate interactions, and blue arrows denote growth-related effects.

The results indicate that overexpression of the *Lst8* gene enhances intracellular energy levels by promoting chloroplast development, thereby increasing photosynthesis and sugar production. It also regulates sugar transport via ABC transporter proteins, facilitating the movement of sugars from chloroplasts to the cytoplasm, and integrates signals from photosynthesis, sugar metabolism, and energy supply to regulate cellular energy metabolism, and it then activates MAPK signaling as well as downstream TOR signaling pathways (Figure 7). TOR senses photosynthesis-derived sugar products (e.g., glucose and sucrose) as well as ATP levels, promoting cell proliferation and growth under sufficient energy conditions.

## 4. Discussion

### 4.1. Role and Effect of Lst8 Gene on Photosynthesis in N. gaditana

The TOR signaling pathway is a key regulator of growth and metabolism in eukaryotic organisms, integrating signals from ambient light and nutrients to optimize photosynthetic efficiency [27,47]. It does so by regulating the synthesis of ribosomal proteins, chlorophyll biosynthesis, and carbon-fixation-related enzymes, ultimately maintaining a balance between anabolic and catabolic processes [48,49,50]. As a crucial component of the TOR complex, LST8 plays a significant role in stabilizing and activating TOR kinase activity [51].

In *N. gaditana*, the interaction between LST8 and the TOR complex may influence the cellular response to nutrient availability, thereby affecting processes such as photosynthesis. In this study, overexpression of *Lst8* enhanced TOR activity, leading to the upregulation of chlorophyll synthesis and other components of the photosynthetic apparatus, thereby increasing the yield of photosynthetic products. In contrast, knockdown of *Lst8* in other microalgae has been shown to inhibit TOR signaling, reducing growth and photosynthetic efficiency, particularly under nutrient-limited conditions [23]. Since LST8 proteins are components of the TORC1 and TORC2 complexes, their regulation may influence lipid metabolism and stress responses in photosynthetic cells. In particular, enhanced TOR signaling via LST8 could lead to more efficient resource allocation for chloroplast development and function, thereby increasing photosynthetic capacity. Additionally, LST8-mediated TOR signaling has been shown to regulate autophagy, aiding in the degradation of damaged organelles and recycling of components during nutrient deprivation [40]. This process helps maintain photosynthetic efficiency, suggesting that TOR signaling, mediated by LST8, plays a key role in regulating cellular energy and carbon allocation. This regulation may be crucial for optimizing photosynthesis and maintaining high growth rates and biomass production in *N. gaditana* under varying environmental conditions, positioning it as a promising candidate for biofuel applications [8,52]. Further studies are needed to clarify how the LST8 protein mediates the regulation between TOR signaling and photosynthesis, particularly the mechanisms involved in light perception, carbon fixation, and photosynthetic protein synthesis. 

### 4.2. Overexpression of the Lst8 Gene Promotes Energy Conversion and Activates TOR Signaling, Affecting Energy Partitioning Homeostasis in N. gaditana

The TOR pathway is a key regulator of cellular metabolism, integrating nutrient signals to modulate growth, development, and energy balance. The TOR complex regulates anabolic processes, such as protein synthesis, lipid metabolism [53,54], and photosynthesis [55,56], with the LST8 protein playing a crucial role in stabilizing the complex. 

In our study, increased *Lst8* expression led to the activation of TOR signaling, which in turn enhanced photosynthetic activity and biomass production. This suggests that *Lst8* plays a pivotal role in optimizing the allocation of cellular energy resources, particularly by influencing metabolic pathways that govern energy storage, sugar metabolism, and protein synthesis. The activation of TORC1 likely promotes a shift in energy partitioning, directing more energy toward growth and photosynthesis, while maintaining metabolic homeostasis under varying environmental conditions [57]. Promoting TOR signaling to upregulate chlorophyll biosynthesis and carbon fixation improves overall energy conversion, enhances cell growth and biomass accumulation, and increases the production of commercially valuable compounds, such as lipids and proteins [4,58]. Additionally, transcriptome analysis indicated that elevated *Lst8* levels trigger the activation of ABC transporters and the MAPK signaling pathway, which may further fine-tune the transmembrane transport of sugars and other metabolites, ensuring an efficient energy distribution across cellular processes. This highlights *Lst8* as a critical mediator in balancing energy conversion and metabolic flux, supporting the idea that manipulating TOR signaling could be a promising strategy for enhancing energy efficiency and productivity in microalgae.

Manipulating energy allocation through *Lst8* overexpression can enhance the production of specific metabolites. Under nutrient-rich conditions, this approach can rapidly promote biomass accumulation, while under nutrient-limited conditions, controls in TOR signaling can be leveraged to enhance lipid accumulation [8,32]. Although there is a risk of disrupting energy homeostasis, the ability to control energy allocation by regulating *Lst8* expression could improve the adaptability and efficiency of *N. gaditana* as a production platform, presenting significant potential for advancing algal biotechnology and improving the economic viability of algal biofuels [8,59]. By enhancing metabolic control through TOR signaling, this strategy could be pivotal in improving the economic viability of algal biofuels, positioning *N. gaditana* as a more efficient and sustainable platform for biofuel production.

## 5. Conclusions

This study demonstrates that overexpression of the *Lst8* gene in *N. gaditana* positively impacts photosynthetic growth by enhancing TOR signaling. The NgLst8 transgenic strain showed increased TOR-related kinase activity, contributing to improved photosynthetic efficiency and growth. Additionally, transcriptome analysis revealed that elevated *Lst8* abundance activates ABC transporter proteins and the MAPK signaling pathway, facilitating the integration of photosynthesis, sugar metabolism, and energy signaling. These findings highlight *Lst8* as a crucial regulator that connects TOR signaling with key metabolic processes in algae, providing valuable insights into optimizing photosynthetic productivity through genetic engineering in *N. gaditana* and potentially other microalgae.

## Figures and Tables

**Table 1:** Primer list used in the PCR test.

Name	Sequences
Lst8-F	CACACTCTAAACCCCAATAAAATGTCATCGGCCGTGGTCCTG
Lst8-R	CTTATCGTCGTCATCCTTGTAATCTATCTGTGCATCGTTCAGTGCC
PHsp-Lst8-1F	GCGGTGCTTGTGTCTGAGGAA
PHsp-Lst8-1R	GCTCGTAGATGGCGAAGATGGT
PHsp-zeo-1F	ATGGCCAAGTTGACCAGTGC
PHsp-zeo-1R	GGTTCAGTCCTGCTCCTCGG

## Data Availability

The raw sequence data reported in this paper were deposited in the Genome Sequence Archive (Genomics, Proteomics & Bioinformatics 2021) in National Genomics Data Center (Nucleic Acids Res 2022), China National Center for Bioinformation/Beijing Institute of Genomics, Chinese Academy of Sciences (GSA: CRA020402), and are publicly accessible at https://ngdc.cncb.ac.cn/gsa; accessed on 13 November 2024.

## Supplementary Materials

The following supporting information can be downloaded at: https://www.mdpi.com/article/10.3390/microorganisms12122574/s1, Figure S1. (A) Phylogenetic tree of gene *Lst8* from different eukaryotic species. (B) Multi-sequence alignment analysis; Figure S2. (A) Chlorophyll fluorescence of WT and NgLst8. (B) Photosynthetic conversion efficiency of WT and NgLst8; Figure S3. (A) TFA content of WT and NgLst8. (B) Carbohydrate content of WT and NgLst8. (C) Lipid yield on day 12 of WT and NgLst8; Figure S4. (A) Heatmap of expression correlation of WT and NgLst8. (B) Principal Component Analysis (PCA). (C) DEG Number of WT and NgLst8. (D) Volcano of differentially genes in WT and NgLst8; Figure S5. Gene expression levels of differentially expressed genes both in the real time PCR and RNA-seq data; Table S1. Summary of the RNA-seq data; Table S2. Significant enrichment of up-regulated DEGs in the pathway; Table S3. The primer sequences used in qRT-PCR.

## References

[1] Burkart G.M., Brandizzi F. (2021). A Tour of TOR Complex Signaling in Plants. Trends Biochem. Sci..

[2] Montane M.H., Menand B. (2019). TOR inhibitors: From mammalian outcomes to pharmacogenetics in plants and algae. J. Exp. Bot..

[3] Albert V., Hall M.N. (2015). mTOR signaling in cellular and organismal energetics. Curr. Opin. Cell Biol..

[4] Couso I., Evans B.S., Li J., Liu Y., Ma F., Diamond S., Allen D.K., Umen J.G. (2016). Synergism between Inositol Polyphosphates and TOR Kinase Signaling in Nutrient Sensing, Growth Control, and Lipid Metabolism in Chlamydomonas. Plant Cell.

[5] Loewith R., Hall M.N. (2011). Target of rapamycin (TOR) in nutrient signaling and growth control. Genetics.

[6] Saxton R.A., Sabatini D.M. (2017). mTOR Signaling in Growth, Metabolism, and Disease. Cell.

[7] Calo G., De Marco M.A., Salerno G.L., Martinez-Noel G.M.A. (2022). TOR signaling in the green picoalga *Ostreococcus tauri*. Plant Sci..

[8] Prioretti L., Carriere F., Field B., Avilan L., Montane M.H., Menand B., Gontero B. (2020). Targeting TOR signaling for enhanced lipid productivity in algae. Biochimie.

[9] Dong P., Xiong F., Que Y., Wang K., Yu L., Li Z., Ren M. (2015). Expression profiling and functional analysis reveals that TOR is a key player in regulating photosynthesis and phytohormone signaling pathways in Arabidopsis. Front. Plant Sci..

[10] Xiong Y., Sheen J. (2015). Novel links in the plant TOR kinase signaling network. Curr. Opin. Plant Biol..

[11] Wullschleger S., Loewith R., Hall M.N. (2006). TOR signaling in growth and metabolism. Cell.

[12] Song Y., Alyafei M.S., Masmoudi K., Jaleel A., Ren M. (2021). Contributions of TOR Signaling on Photosynthesis. Int. J. Mol. Sci..

[13] Yang Q., Inoki K., Ikenoue T., Guan K.L. (2006). Identification of Sin1 as an essential TORC2 component required for complex formation and kinase activity. Genes. Dev..

[14] Maegawa K., Takii R., Ushimaru T., Kozaki A. (2015). Evolutionary conservation of TORC1 components, TOR, Raptor, and LST8, between rice and yeast. Mol. Genet. Genom..

[15] Díaz-Troya S., Florencio F.J., Crespo J.L. (2008). Target of Rapamycin and LST8 Proteins Associate with Membranes from the Endoplasmic Reticulum in the Unicellular Green Alga *Chlamydomonas reinhardtii*. Eukaryot. Cell.

[16] Shi L., Wu Y., Sheen J. (2018). TOR signaling in plants: Conservation and innovation. Development.

[17] Zhao Y., Wang X.Q. (2020). The hot issue: TOR signalling network in plants. Funct. Plant Biol..

[18] Moreau M., Azzopardi M., Clement G., Dobrenel T., Marchive C., Renne C., Martin-Magniette M.L., Taconnat L., Renou J.P., Robaglia C. (2012). Mutations in the Arabidopsis homolog of LST8/GbetaL, a partner of the target of *Rapamycin kinase*, impair plant growth, flowering, and metabolic adaptation to long days. Plant Cell.

[19] Forzani C., Duarte G.T., Van Leene J., Clement G., Huguet S., Paysant-Le-Roux C., Mercier R., De Jaeger G., Leprince A.S., Meyer C. (2019). Mutations of the AtYAK1 Kinase Suppress TOR Deficiency in Arabidopsis. Cell Rep..

[20] Pancha I., Shima H., Higashitani N., Igarashi K., Higashitani A., Tanaka K., Imamura S. (2019). Target of rapamycin-signaling modulates starch accumulation via glycogenin phosphorylation status in the unicellular red alga *Cyanidioschyzon merolae*. Plant J..

[21] Swer P.B., Mishra H., Lohia R., Saran S. (2016). Overexpression of TOR (target of rapamycin) inhibits cell proliferation in *Dictyostelium discoideum*. J. Basic Microbiol..

[22] Perez-Perez M.E., Couso I., Crespo J.L. (2017). The TOR Signaling Network in the Model Unicellular Green Alga *Chlamydomonas reinhardtii*. Biomolecules.

[23] Couso I., Perez-Perez M.E., Ford M.M., Martinez-Force E., Hicks L.M., Umen J.G., Crespo J.L. (2020). Phosphorus Availability Regulates TORC1 Signaling via LST8 in Chlamydomonas. Plant Cell.

[24] Jang H., Ehrenreich I.M. (2012). Genome-wide characterization of genetic variation in the unicellular, green alga *Chlamydomonas reinhardtii*. PLoS ONE.

[25] Upadhyaya S., Agrawal S., Gorakshakar A., Rao B.J. (2020). TOR kinase activity in *Chlamydomonas reinhardtii* is modulated by cellular metabolic states. FEBS Lett..

[26] Tsuji Y., Ishikawa T. (2024). Assessing Target of Rapamycin (TOR) activity in the diatom *Phaeodactylum tricornutum* using commercially available materials. Algal Res..

[27] Mallen-Ponce M.J., Perez-Perez M.E., Crespo J.L. (2022). Photosynthetic assimilation of CO_2_ regulates TOR activity. Proc. Natl. Acad. Sci. USA.

[28] Arias C., Obudulu O., Zhao X., Ansolia P., Zhang X., Paul S., Bygdell J., Pirmoradian M., Zubarev R.A., Samuelsson G. (2020). Nuclear proteome analysis of Chlamydomonas with response to CO_2_ limitation. Algal Res..

[29] Xu C., Min J. (2011). Structure and function of WD40 domain proteins. Protein Cell.

[30] Jain B.P., Pandey S. (2018). WD40 Repeat Proteins: Signalling Scaffold with Diverse Functions. Protein J..

[31] Yue Z., Lv Z., Shao Y., Zhang W., Zhao X., Guo M., Li C. (2019). Cloning and characterization of the target protein subunit lst8 of rapamycin in *Apostichopus japonicus*. Fish Shellfish Immunol..

[32] Takeuchi T., Benning C. (2019). Nitrogen-dependent coordination of cell cycle, quiescence and TAG accumulation in Chlamydomonas. Biotechnol. Biofuels.

[33] Li F., Gao D., Hu H. (2014). High-efficiency nuclear transformation of the oleaginous marine *Nannochloropsis* species using PCR product. Biosci. Biotechnol. Biochem..

[34] Chen Y., Hu H. (2019). High efficiency transformation by electroporation of the freshwater alga *Nannochloropsis limnetica*. World J. Microbiol. Biotechnol..

[35] Livak K.J., Schmittgen T.D. (2001). Analysis of Relative Gene Expression Data Using Real-Time Quantitative PCR and the 2^−ΔΔCT^ Method. Methods.

[36] Jassby A.D., Platt T. (2003). Mathematical formulation of the relationship between photosynthesis and light for phytoplankton. Limnol. Oceanogr..

[37] Chini Zittelli G., Rodolfi L., Biondi N., Tredici M.R. (2006). Productivity and photosynthetic efficiency of outdoor cultures of *Tetraselmis suecica* in annular columns. Aquaculture.

[38] Chen G.H., Liu M.J., Xiong Y., Sheen J., Wu S.H. (2018). TOR and RPS6 transmit light signals to enhance protein translation in deetiolating *Arabidopsis seedlings*. Proc. Natl. Acad. Sci. USA.

[39] Xiong Y., McCormack M., Li L., Hall Q., Xiang C., Sheen J. (2013). Glucose-TOR signalling reprograms the transcriptome and activates meristems. Nature.

[40] Chung T. (2019). How phosphoinositides shape autophagy in plant cells. Plant Sci..

[41] Delage E., Puyaubert J., Zachowski A., Ruelland E. (2013). Signal transduction pathways involving phosphatidylinositol 4-phosphate and phosphatidylinositol 4,5-bisphosphate: Convergences and divergences among eukaryotic kingdoms. Prog. Lipid Res..

[42] Marat A.L., Haucke V. (2016). Phosphatidylinositol 3-phosphates-at the interface between cell signalling and membrane traffic. EMBO J..

[43] Morales-Herrera S., Paul M.J., Van Dijck P., Beeckman T. (2024). SnRK1/TOR/T6P: Three musketeers guarding energy for root growth. Trends Plant Sci..

[44] Gobel M., Fichtner F. (2023). Functions of sucrose and trehalose 6-phosphate in controlling plant development. J. Plant Physiol..

[45] Yang A., Suh W.I., Kang N.K., Lee B., Chang Y.K. (2018). MAPK/ERK and JNK pathways regulate lipid synthesis and cell growth of *Chlamydomonas reinhardtii* under osmotic stress, respectively. Sci. Rep..

[46] Gomez-Osuna A., Calatrava V., Galvan A., Fernandez E., Llamas A. (2020). Identification of the MAPK Cascade and its Relationship with Nitrogen Metabolism in the Green Alga *Chlamydomonas reinhardtii*. Int. J. Mol. Sci..

[47] Bakshi A., Moin M., Madhav M.S., Datla R., Kirti P.B. (2021). Target of Rapamycin (TOR) negatively regulates chlorophyll degradation and lipid peroxidation and controls responses under abiotic stress in *Arabidopsis thaliana*. Plant Stress.

[48] Dobrenel T., Marchive C., Sormani R., Moreau M., Mozzo M., Montane M.H., Menand B., Robaglia C., Meyer C. (2011). Regulation of plant growth and metabolism by the TOR kinase. Biochem. Soc. Trans..

[49] Lutt N., Brunkard J.O. (2022). Amino Acid Signaling for TOR in Eukaryotes: Sensors, Transducers, and a Sustainable Agricultural fuTORe. Biomolecules.

[50] Couso I., Smythers A.L., Ford M.M., Umen J.G., Crespo J.L., Hicks L.M. (2021). Inositol polyphosphates and target of rapamycin kinase signalling govern photosystem II protein phosphorylation and photosynthetic function under light stress in Chlamydomonas. New Phytol..

[51] Chen E.J., Kaiser C.A. (2003). LST8 negatively regulates amino acid biosynthesis as a component of the TOR pathway. J. Cell Biol..

[52] Alboresi A., Perin G., Vitulo N., Diretto G., Block M., Jouhet J., Meneghesso A., Valle G., Giuliano G., Marechal E. (2016). Light Remodels Lipid Biosynthesis in *Nannochloropsis gaditana* by Modulating Carbon Partitioning between Organelles. Plant Physiol..

[53] Werth E.G., McConnell E.W., Couso Lianez I., Perrine Z., Crespo J.L., Umen J.G., Hicks L.M. (2019). Investigating the effect of target of rapamycin kinase inhibition on the *Chlamydomonas reinhardtii* phosphoproteome: From known homologs to new targets. New Phytol..

[54] Wu Y., Shi L., Li L., Fu L., Liu Y., Xiong Y., Sheen J. (2019). Integration of nutrient, energy, light, and hormone signalling via TOR in plants. J. Exp. Bot..

[55] Ingargiola C., Turqueto Duarte G., Robaglia C., Leprince A.S., Meyer C. (2020). The Plant Target of Rapamycin: A Conduc TOR of Nutrition and Metabolism in Photosynthetic Organisms. Genes.

[56] Juppner J., Mubeen U., Leisse A., Caldana C., Wiszniewski A., Steinhauser D., Giavalisco P. (2018). The target of rapamycin kinase affects biomass accumulation and cell cycle progression by altering carbon/nitrogen balance in synchronized *Chlamydomonas reinhardtii* cells. Plant J..

[57] Li L., Zhu T., Huang L., Ren M. (2022). Target of Rapamycin Signaling Involved in the Regulation of Photosynthesis and Cellular Metabolism in *Chlorella sorokiniana*. Int. J. Mol. Sci..

[58] Bakshi A., Moin M., Kumar M.U., Reddy A.B., Ren M., Datla R., Siddiq E.A., Kirti P.B. (2017). Ectopic expression of Arabidopsis Target of Rapamycin (AtTOR) improves water-use efficiency and yield potential in rice. Sci. Rep..

[59] Imamura S., Kawase Y., Kobayashi I., Shimojima M., Ohta H., Tanaka K. (2016). TOR (target of rapamycin) is a key regulator of triacylglycerol accumulation in microalgae. Plant Signal Behav..

