# Supplementary material for: NgLst8 Coactivates TOR Signaling to Activate Photosynthetic Growth in Nannochloropsis gaditana
Source: Microorganisms. 2024 Dec 13;12(12):2574. doi: 10.3390/microorganisms12122574 (PMC11678606; doi:10.3390/microorganisms12122574)
Supplement: Supplementary file 1 [file microorganisms-12-02574-s001.zip › microorganisms-3345002-supplementary.pdf]

Supplementary data

Supplementary Table S1. Summary of the RNA-seq data

Sample	Clean reads	Clean bases	GC Content	Clean Reads Q20(%)	Clean Reads Q30(%)
C1	22243081	6666396838	56.41	98.15	93.82
C2	21773002	6524736652	56.33	97.8	92.73
C3	21485515	6436461214	56.51	98.16	93.82
Nglst8-1	22312437	6683461499	56.18	98.11	93.6
Nglst8-2	22240585	6663273537	56.16	97.86	92.71
Nglst8-3	21905069	6563510955	56.22	98.07	93.47

Supplementary Table S2. Significant enrichment of up-regulated DEGs in the pathway

Gene ID	Log2(Fold change)	FDR	regulated	Description
**ABC transporters**				
gene-Naga_102378g1	1.420862052	9.93E-08	up	ATP-binding cassette, subfamily A (ABC1), member 3
gene-Naga_100055g35	2.136857088	4.14E-07	up	ATP-binding cassette, subfamily G (WHITE), member 2
gene-Naga_100870g2	1.550056008	4.88E-09	up	ATP-binding cassette, subfamily B (MDR/TAP), member 1
gene-Naga_100093g1	1.01261893	0.00024	up	ATP-binding cassette, subfamily A (ABC1), member 3
gene-Naga_100051g26	2.034688283	9.70E-08	up	ATP-binding cassette, subfamily G (WHITE), member 2
gene-Naga_100079g18	3.024500056	4.60E-16	up	ATP-binding cassette, subfamily G (WHITE), member 2
gene-Naga_102427g1	1.258380747	0.001302	up	ATP-binding cassette, subfamily A (ABC1), member 3
gene-Naga_100157g12	2.444072195	7.39E-08	up	ATP-binding cassette, subfamily G (WHITE), member 2
gene-Naga_102431g1	1.384317056	2.45E-07	up	ATP-binding cassette, subfamily A (ABC1), member 3
**MAPK signaling pathway - plant**				
gene-Naga_100243g2	2.634739411	1.82E-06	up	mitogen-activated protein kinase 6
gene-Naga_100001g218	1.309972225	1.26E-06	up	mitogen-activated protein kinase 6
gene-Naga_100011g59	1.039067295	5.40E-05	up	mitogen-activated protein kinase 6
gene-Naga_100045g17	3.002286449	2.17E-134	up	mitogen-activated protein kinase kinase 3
gene-Naga_100045g16	1.372753109	2.99E-08	up	mitogen-activated protein kinase kinase 3
**Starch and sucrose metabolism**				
NewGene_473	1.617042	1.30E-20	up	fructokinase
gene-Naga_100045g12	2.417166	3.70E-30	up	endoglucanase

gene-Naga_100014g5	1.586714	0.000167311	up	endoglucanase
gene-Naga_100079g22	3.516247	6.58E-25	up	cellulose synthase (UDP-forming)
**Fatty acid biosynthesis**				
gene-Naga_100028g49	1.806089368	5.77E-16	up	fatty acid synthase, animal type
gene-Naga_100007g48	1.450494414	2.94E-26	up	fatty acid synthase, animal type
gene-Naga_101811g1	1.087447644	2.92E-06	up	fatty acid synthase, animal type
gene-Naga_102202g1	1.318600931	6.89E-05	up	[acyl-carrier-protein] S-malonyltransferase
**Betalain biosynthesis**				
gene-Naga_100010g61	1.306633674	0.000222607	up	tyrosinase
**Autophagy - other**				
gene-Naga_100016g80	4.35449259	2.59E-23	up	serine/threonine-protein kinase ULK2
gene-Naga_100690g2	2.030718767	9.77E-07	up	serine/threonine-protein kinase mTOR

Supplementary Table S3. The primer sequences used in qRT-PCR

Primer Name	Primer sequence
Lst8-F	GTCACTACCTCAGCAGATAA
Lst8-R	CTAAGTTGGAGCCTTGATAAG
Naga_100027g19（LHCP5）F	ACCATGAAGTTGGGCTCCTCT
Naga_100027g19（LHCP5） R	GCTTTGGGTTGTTGGTGAATC
Naga_100012g50（VCP1） F	CGAGAAGTTCAACCGTTACCG
Naga_100012g50（VCP1） R	GAGGTGATGGCGTTGATGC
Naga_100173g12 （LHCSR1） F	TCAACGAGAAGGAGATGGATGGT
Naga_100173g12 （LHCSR1） R	AGATGGTGCGAGCCTCAATG
Naga_100641g3 （LHCr7） F	GCCTGTCAGAGATCAACGAAGTC
Naga_100641g3 （LHCr7） R	CGGTCCAGAACGCATCCATT
Naga_100056g42 （LHCA） F	CCTGCTCGGCTCTTGTTCTG
Naga_100056g42 （LHCA） R	AGTGAGTGTGAGGTACTGTGGT
Naga_100168g14 （LHCP29） F	GCCTGTGGGTCTCTGAGTTC
Naga_100168g14 （LHCP29） R	ATTCTCCGTGTCCTGCTTTCC
Naga_101891g1 （FBP） F	CTAGAATGCCCACCATGACCA
Naga_101891g1 （FBP） R	CTGGATTGAGGAAAGGAGCACT
Naga_100154g7 （ALDO） F	CAGTCACACTCCCATAATCCTTCA
Naga_100154g7 （ALDO） R	GCAATCTTCTTGGCGGTCTCA
Naga_100410g3 （PGK） F	ATGACCACAGTTCCGAAGAAGATG
Naga_100410g3 （PGK） R	GAAGAAGGAGGGAGCGACAAAG
Naga_100081g16 (GAPDH) F	GCCTTGCTCCCATCGTCAA
Naga_100081g16 (GAPDH) R	AGCCGTCCACAACCATCTG
Naga_100049g1 （SBP） F	GGCAAGGGTGTCTTCGTCAA
Naga_100049g1 （SBP） R	TGCGGTCCTCGGTCTTGTA
Naga_100157g10 （PRK） F	GCCTTAATCGTCCATCTGTTAGC
Naga_100157g10 （PRK） R	TCAAGACTGCCGCCGTAA
Naga_100022g28 (PI4KB) F	TCCGAGTCCTTCGCCTTCT
Naga_100022g28 (PI4KB) R	CATCACCTGCTTCCACCACTT
Naga_100007g20 （PIP5K） F	CGCCACTATCGCACAAGAAG
Naga_100007g20 （PIP5K） R	TGTCGGTTGCTCGTGTATAAAG
Naga_100084g15 （PIK3C3） F	CGGCGGTTGTCTTGGAGAA
Naga_100084g15 （PIK3C3） R	GCAACTGAGAAGGACGAACTGAA
Naga_101571g1 （PPIP5K） F	CGAAACTCGCACTGAGCAATC
Naga_101571g1 （PPIP5K） R	CGGTGGAGGTGGAAAGAAC
Naga_100199g9 （CDIPT） F	TGCCTGTGTATGCTGGATATTGG
Naga_100199g9 （CDIPT） R	GCCTCCTCCGACTTGTGATG
Naga_100056g35（CDS1） F	GTAGGCAAGATACTGCACAAGC
Naga_100056g35（CDS1） R	AGCTATTGGACCGAGGACTGAG
Naga_100064g19 （IPMK） F	GCAATCCTCGTCGCTACCTT
Naga_100064g19 （IPMK） R	CGGACAATGACAGAAGCCATCA
Naga_100041g12（IMPL2）F	CAGACTGCCGCCTCATCTT
Naga_100041g12（IMPL2）R	GCCACGAAGGAGTTCACAGA
Naga_100024g53（CASKIN）F	AGGACAAGAGCGGGCAAAC
Naga_100024g53（CASKIN）R	AAGCATAGAGTGGAGGACAAGGA
Naga_100089g7（dgkA）F	ACAAGTTCTACTACGCCCTGATG

Naga_100089g7（dgkA）R	ATTGATGTTGAGGACGATGAAGGA
Naga_100045g12 F	CCGCTGGAATTTGTTTGCCA
Naga_100045g12 R	GCCCTTATACTGCGGATGCT
Naga_100079g22 F	ATTTCTGGGCGGGTCTTCAG
Naga_100079g22 R	ATCAGCGAGGGCGATAAAGG
Naga_100055g35 F	ACGCGATTGGTCCTTACCTG
Naga_100055g35 R	AAACTTGCCGATGTCCGAGT
Naga_100045g17 F	CTGGTCTTTCGTCGGGTTCA
Naga_100045g17 R	GTGCCTTTCGCGTTGACTTT
Naga_100243g2 F	CGTCTCGCCTCGCTTTCTAA
Naga_100243g2 R	AAATTCCGTGCCCTCGATGA
Naga_100028g49 F	TCGACGCTTGCCACTTTTTG
Naga_100028g49 R	GAGGCGAAGACGGTGAAGAA
Naga_100007g48 F	AGGGGGCCTATGTTGGAGAT
Naga_100007g48 R	AACCATGTCGGGGAGTTCAC
Naga_100249g6 F	CGCTCTCGGTTCTACTTACTGAAG
Naga_100249g6 R	ACATCGGATAGCACGCACAG
Naga_100026g54 F	CGAAGGCACGACGCTAAC
Naga_100026g54 R	ACCGCATTATTGTGTAGGCTATGA
Naga_100322g6 F	GGAGATGAAGGTGCTGACGAA
Naga_100322g6 R	GAACACGGTGGCGAGGAT
Naga_100010g84 F	CTGACGACGGATGTGTTGGT
Naga_100010g84 R	ATTCCAGCAGCACTTCTTCCA
Naga_100039g18 F	GCACGGCATCAGCAGAAC
Naga_100039g18 R	ACGGAGCAGCAAGAGGTT

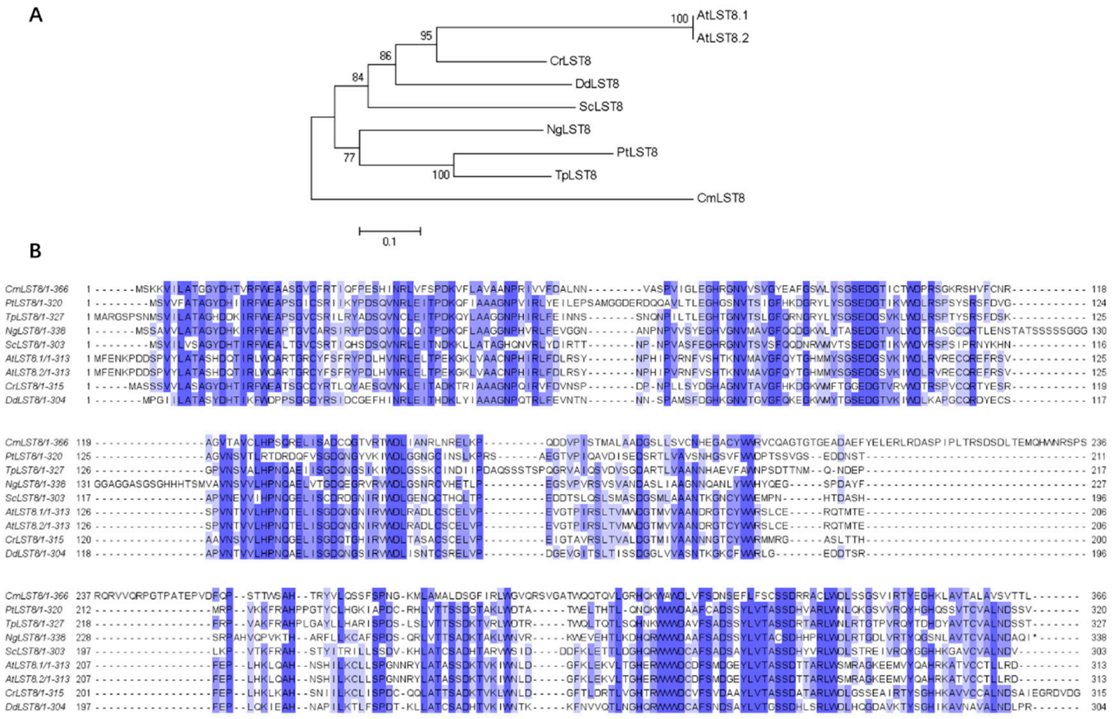

Supplementary Figure S1. (A) Phylogenetic tree of gene *Lst8* from different eukaryotic species. (B) Multi-sequence alignment analysis. Analysis using InterPro (InterPro (ebi.ac.uk)).

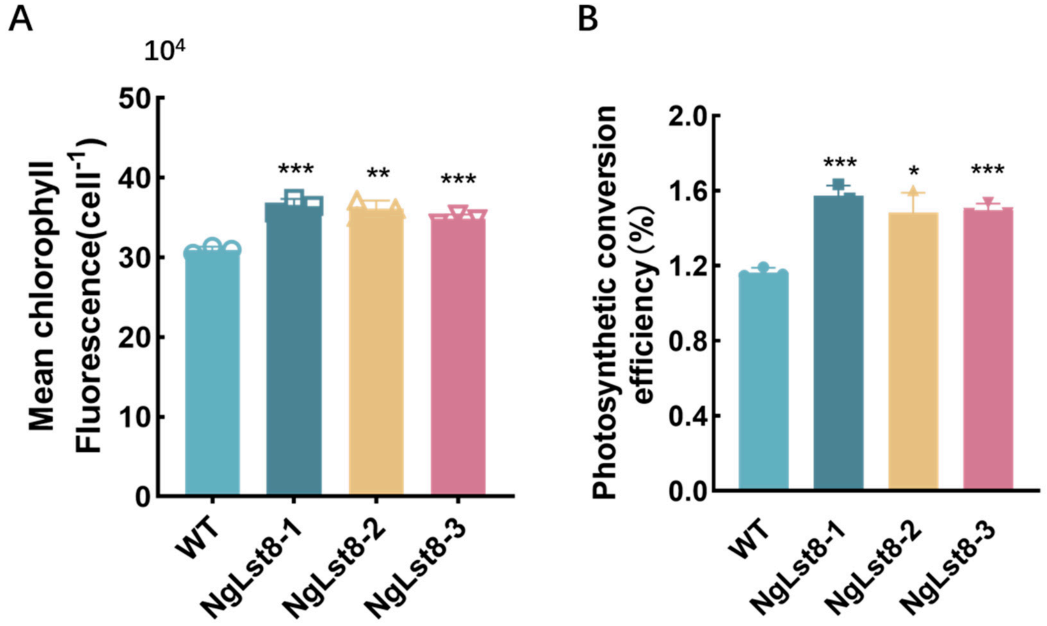

Supplementary Figure S2. (A) Chlorophyll fluorescence of WT and NgLst8. (B) Photosynthetic conversion efficiency of WT and NgLst8. ns indicates $p > 0.05$, * $p < 0.05$, ** $p < 0.01$, *** $p < 0.001$. Error bars represent standard deviations calculated from three independent biological replicates. All data were analyzed through two-way ANOVA (GraphPad Prism 8.4).

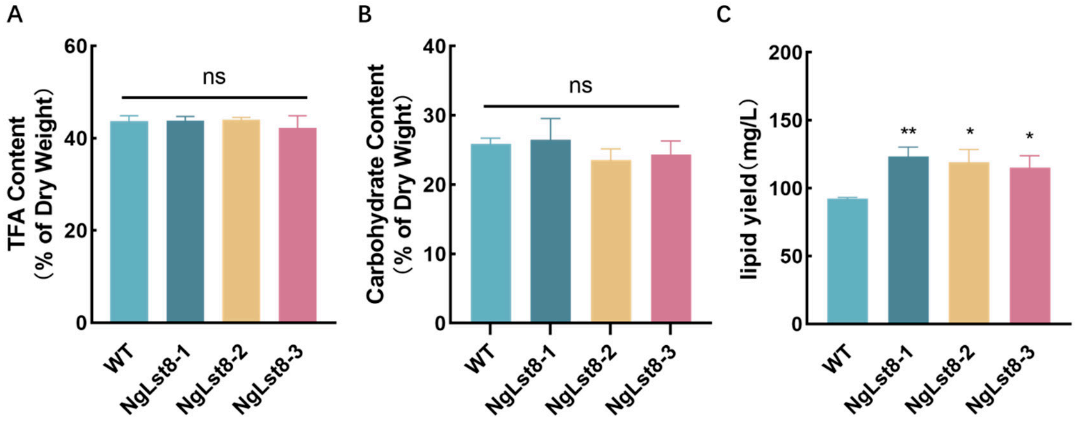

Supplementary Figure S3. (A) TFA content of WT and NgLst8. (B) Carbohydrate content of WT and NgLst8. (C) Lipid yield on day 12 of WT and NgLst8.

ns indicates p > 0.05, * p < 0.05, ** p < 0.01. Error bars represent standard deviations calculated from three independent biological replicates. All data were analyzed through two-way ANOVA (GraphPad Prism 8.4).

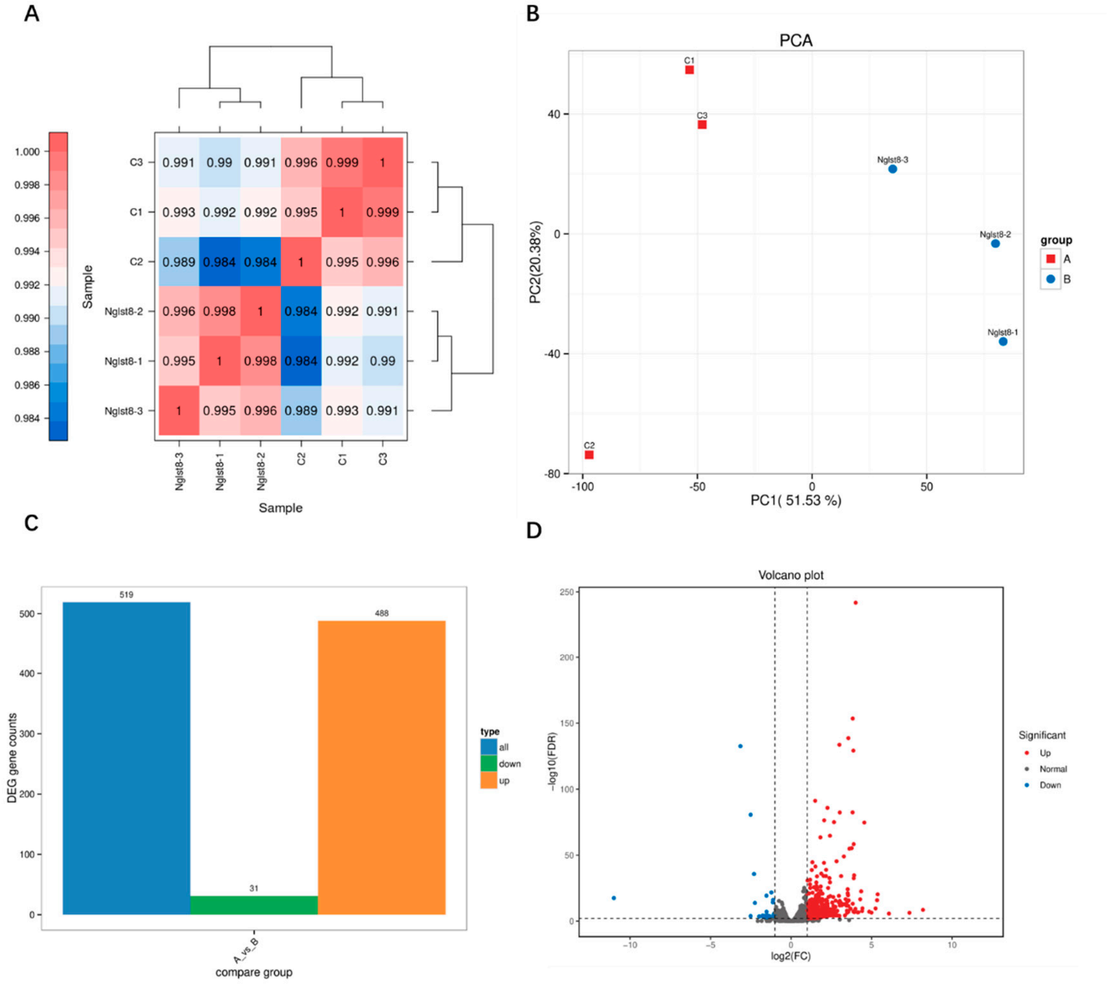

Supplementary Figure S4. (A) Heatmap of expression correlation of WT and NgLst8. (B) Principal Component Analysis (PCA). (C) DEG Number of WT and NgLst8. (D) Volcano of differentially genes in WT and NgLst8.

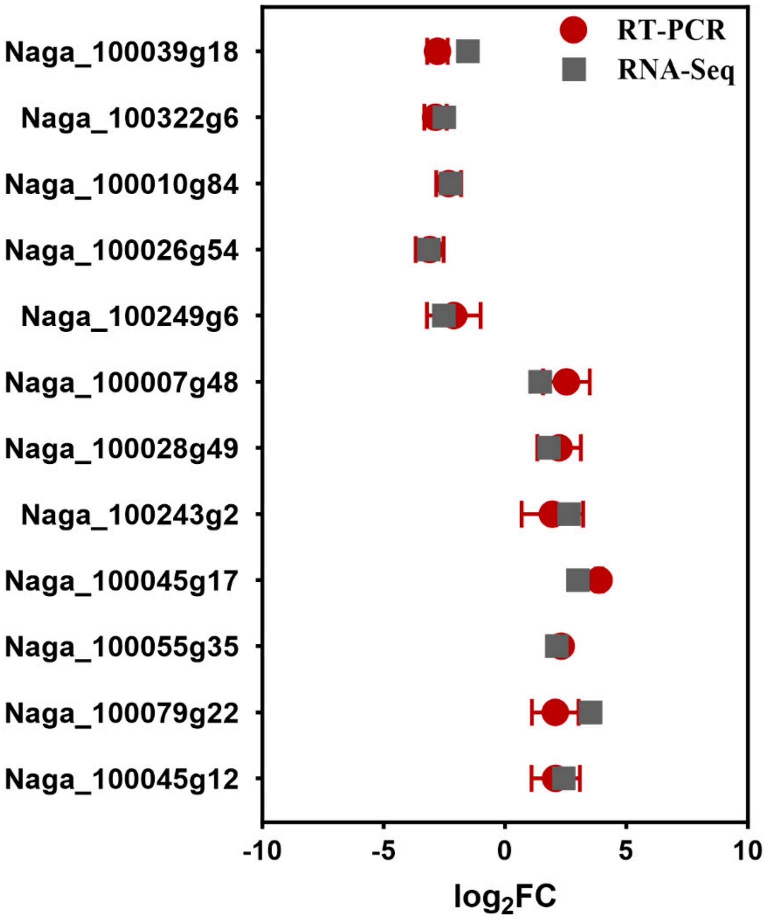

Supplementary Figure S5. Gene expression levels of differentially expressed genes both in the real time PCR and RNA-seq data. Error bars represent standard deviations calculated from three independent biological replicates.
